# Effectiveness and Safety of Acupotomy for Lumbar Disc Herniation: A Randomized, Assessor-Blinded, Controlled Pilot Study

**DOI:** 10.1155/2018/5871657

**Published:** 2018-08-06

**Authors:** So Yun Kim, Eunseok Kim, Ojin Kwon, Chang-Hyun Han, Young-Il Kim

**Affiliations:** ^1^Department of Acupuncture and Moxibustion Medicine, Daejeon University Dunsan Korean Medicine Hospital, Daejeon 35235, Republic of Korea; ^2^Department of Acupuncture & Moxibustion Medicine, College of Korean Medicine, Daejeon University, 62 Daehak-ro, Dong-gu, Daejeon 34520, Republic of Korea; ^3^Clinical Research Division, Korea Institute of Oriental Medicine, Daejeon 34054, Republic of Korea

## Abstract

**Objective:**

Patients with lumbar disc herniation (LDH) suffer from pain, physical disabilities, and low quality of life. This study was designed to evaluate the effectiveness and safety of acupotomy in patients with LDH.

**Method:**

Fifty participants with LDH were recruited to this randomized, assessor-blinded, controlled study and randomly assigned to the acupotomy (n = 25) or manual acupuncture (n = 25) group. The acupotomy group received acupotomy four times in 2 weeks, while the manual acupuncture group received manual acupuncture six times in 2 weeks. The follow-up visit was planned in the 4th week (i.e., 2 weeks after the final intervention). The primary outcome was the change in the Visual Analogue Scale (VAS) at follow-up. The changes in the Oswestry Disability Index (ODI), Modified-Modified Schober Test (MMST), and EuroQol Five Dimensions (EQ-5D) questionnaire were also evaluated. An intention-to-treat analysis was applied and adverse events were recorded.

**Results:**

The acupotomy group showed significant changes in VAS, ODI, and EQ-5D after intervention. VAS and ODI in the 4th week were lower in the acupotomy than in the manual acupuncture group. The acupotomy group showed consistent changes in VAS and ODI in the 1st, 2nd, and 4th week. No serious adverse event was reported in the acupotomy group.

**Conclusion:**

This study suggests greater therapeutic effects of acupotomy on relieving pain and improving the functional disability associated with LDH than those observed with manual acupuncture.

## 1. Introduction

Lumbar disc herniation (LDH) is a state of displaced intervertebral disc material, including the nucleus pulposus or annulus fibrosis, which can cause low-back pain and/or radicular pain, paresthesia, or weakness in the lower limbs [1, 2]. Current therapies include nonsurgical and surgical methods. The nonsurgical treatments include conservative management and invasive treatments [3, 4]. For the conservative management, nonsteroidal anti-inflammatory drugs (NSAIDs), acetaminophen, muscle relaxants, and steroidal drugs are commonly used among others. Nonpharmacological strategies such as acupuncture, physical therapy, and exercise are also recommended. If the conservative therapy fails to relieve the symptoms, more invasive treatments such as epidural steroid injection can be applied. When these more invasive procedures fail to control the LDH symptoms, subsequent surgeries are considered.

Before considering surgery, it is important to discuss other therapeutic options in patients who received nonsurgical treatments with no success. Epidural injections are effective in pain relief for a short time but may require additional injections because of recurring symptoms [5, 6]. There have been attempts to find effective and safe treatments before surgical procedures for symptomatic LDH [7–9].

As a valuable option in LDH, acupotomy has the characteristics of both acupuncture and surgical procedures [10, 11]. Acupotomy uses a bladed needle composed of a thick, flat-head, and cylindrical body, which is optimized for alleviating the adhesion of a lesion. Acupotomy is used for musculoskeletal pain such as low-back, postneck, knee, and shoulder pain and even in metabolic diseases such as diabetes and lymphatic edema [12–15].

Although the potential benefit of acupotomy in patients with LDH has been previously suggested, more evidence is needed regarding its effectiveness and safety [11, 16, 17]. Therefore, we designed this controlled, pilot study on the effectiveness and safety of acupotomy in patients with LDH compared with manual acupuncture.

## 2. Method

### 2.1. Design and Participants

The study protocol has been previously published [18]. In summary, 50 participants with symptomatic LDH were recruited to this randomized, two parallel-armed, controlled, assessor-blinded, single center, pilot clinical trial. The participants were recruited from the outpatients at Daejeon University Dunsan Korean Medicine Hospital (DUDKMH) from July 11, 2016, to January 20, 2017. The study was approved by the Institutional Review Board of DUDKMH (DJDSKH-16-BM-05) and registered in the Clinical Research Information Service (CRIS) (KCT0002188) in South Korea.

To test the effectiveness of acupotomy in 40 participants (20 per group), a total of 50 participants (25 per group) were recruited after considering the 20% drop-out rate. Patients aged between 20 and 80 years who were diagnosed with LDH and showed symptoms such as low-back pain, radicular pain, and paresthesia in the lower limbs were recruited. The LDH diagnosis was based on medical imaging findings. Patients who were at the bulging stage based on medical images were excluded, and those who were diagnosed with LDH at the protrusion and extrusion stages or above were recruited.

### 2.2. Randomization and Blinding

Participants were randomly assigned to either the acupotomy group (n = 25) or the manual acupuncture group (n = 25). The random number list was generated by an independent statistician. Participants were informed of the possibility of random allocation and of each intervention procedure. Different researchers were in charge of the intervention procedure, outcome assessment data management, and statistical analysis. As the intervention procedure and needle sizes vary distinctively between acupotomy and acupuncture, it was difficult to blind the participants and intervention practitioners. Outcome assessors and data managers were blinded to the allocation status of each participant. Each intervention practitioner performed only one intervention, either acupotomy or acupuncture, and was not involved in the outcome measurements.

### 2.3. Interventions

In acupotomy group, four acupotomy treatments were administered twice per week for 2 weeks using flat-head-screw-driver-shaped stainless steel disposable sterilized acupotomy needles (1.2 mm in diameter and 75 mm in length; Hansung Precision Manufacture, Seoul, South Korea). The insertion points were set at the corresponding disc level based on the medical imaging finding, 20–30 mm away from the spinous process, to the depth of 50–60 mm. They could be inserted on one side or both sides, according to the symptom and appearance of LDH on the medical image, and no more than three acupotomy sites were chosen. After local sterilization with 10% betadine solution and anesthetization with lidocaine, the needle was inserted, manipulated, and removed immediately. Disposable sterilized wet-cuppings were applied on the acupotomy site for 5 minutes to prevent local hematoma [19].

In the manual acupuncture group, six manual acupuncture treatments were administered three times per week for 2 weeks using 0.25 mm × 40 mm, single-use, sterile, stainless steel needles (Dongbang Acupuncture Inc., Chungnam, Republic of Korea). The acupuncture treatment was performed at GV3 and bilateral BL23, BL24, BL25, BL26, GB3, BL40, and BL60 [20]. The depth of insertion was 20 mm for BL40 and BL60 and 30 mm for the other acupuncture points. The needles were removed after 15 minutes.

Acetaminophen was administered to the acupotomy group for rescue medication. Details of the intervention and cointervention have also been published [18].

### 2.4. Outcome Measurements

The primary outcome was the change in the Visual Analogue Scale (VAS) score for low-back pain and/or leg pain between baseline and follow-up visit on the 4^th^ week (2 weeks after the last intervention). The secondary outcome measures included the Korean version of Oswestry Disability Index (ODI) [21], Modified-Modified Schober Test (MMST) [22], and EuroQol Five Dimensions (EQ-5D) questionnaire [23]. All the above outcomes were measured at the screening visit and 1^st^, 2^nd^, and 4^th^ week after randomization.

The adverse events were assessed during each visit based on the vital signs, medical examinations, and other test results. The causal relationships between adverse events and acupotomy or acupuncture intervention and severity of the adverse events were assessed, such as pain, bleeding, hematoma, or bruise.

All the outcome values were registered on the eCRF, which was designed by Korea Institute of Oriental Medicine and only accessible to nonblinded researchers.

### 2.5. Statistical Analysis

Statistical analyses were performed using SAS® software (version 9.1.4, SAS institute, Inc., Cary, NC) by a statistician blinded to the participant allocation. If a participant received the intervention and provided the outcome measurement more than once, the data would be analyzed. The independent sample t-test or Wilcoxon signed-rank test for continuous variables and the chi-squared or Fisher's exact test for categorical variables were used to examine potential differences in baseline demographics and medical history variables between the acupotomy and manual acupuncture groups. The analysis of covariance (ANCOVA) was used to compare the mean changes in VAS, ODI, MMST, and EQ-5D from the baseline to 4^th^ week. The paired t-test or Wilcoxon signed-rank test was used to compare the outcomes before and after treatment within each group. The repeated measures analysis of variance (ANOVA) was used to assess the differences between the two groups over time. A value of P <.05 was considered statistically significant.

## 3. Results

### 3.1. Recruitment and Baseline Data

**Figure 1:**
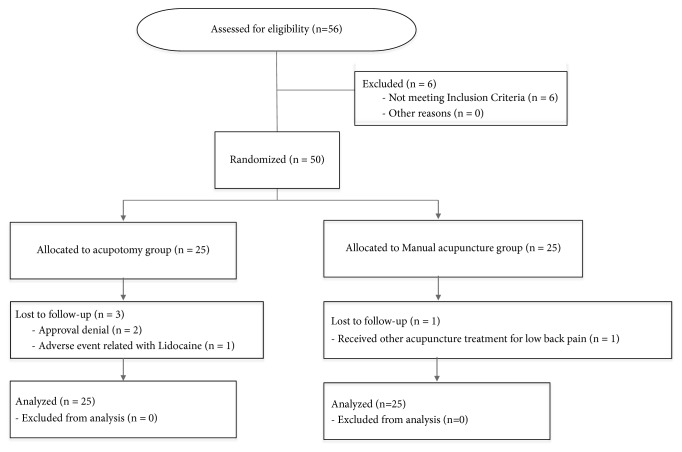
Flow diagram.

Of the 56 participants with LDH who were interviewed during the recruitment period, 50 met the inclusion criteria and were randomly assigned to the acupotomy group (n = 25) and the manual acupuncture control group (n = 25) (Figure 1). Of the 50 participants, 46 completed this study, while the remaining four dropped out. Of the four participants who dropped out, two participants in the acupotomy group withdrew their consent during their participation in the research, and one was withdrawn due to the side effects of lidocaine. One participant in the control group was dropped because of receiving additional acupuncture treatment for back pain at another clinic before completing this trial.

Demographic and medical data like sex, age, weight, height, smoking, drinking, medical history of hypertension, diabetes mellitus, or others and medication use were collected and analyzed. No significant differences in age, sex, weight, height, smoking status, alcohol consumption, medical history, and medication use were found between the two groups (p >.05).

Table 1 shows the mean baseline VAS, MMST, ODI, and EQ-5D for the acupotomy and manual acupuncture group.

### 3.2. Primary Outcome

We compared the change of mean VAS scores between two groups by ANCOVA. The covariate value of the mean VAS score was more significantly reduced in the acupotomy group at the 4^th^ week from the baseline compared with the manual acupuncture group (P <.01) (Table 2).

We also compared the difference in VAS score before and after the intervention within each group by independent sample t-test. In the acupotomy group, the mean VAS score significantly decreased after acupotomy (from 62.74±16.666 to 37.52±24.714, P <.001). In the manual acupuncture group, although the mean VAS score decreased after manual acupuncture, there was no significant difference (from 58.83±16.846 to 52.48±23.216, P >.05) (Table 2).

### 3.3. Secondary Analysis

After the paired t-test of the baseline and 4^th^ week outcomes, other secondary outcomes showed the following results: The mean MMST score significantly increased after acupotomy (from 4.69±1.786 to 5.48±1.581, P <.05), whereas it did not increase significantly after manual acupuncture (from 4.84±1.348 to 5.01±1.304, P >.05). The mean ODI score significantly decreased after acupotomy (from 36.81±11.678 to 27.24±12.302, P <.001), whereas it did not decrease significantly after manual acupuncture (from 30.84±13.641 to 30.76±14.078, P >.05). The mean EQ-5D score significantly increased after both acupotomy (from 0.779±0.121 to 0.792±0.114, P <.05) and manual acupuncture (from 0.737±0.112 to 0.757±0.111, P <.001) (Table 2).

We performed ANCOVA to compare the change in the secondary outcomes between the two groups from the baseline to 4th week. The covariate value of the mean ODI score was more significantly reduced in the acupotomy group at the 4th week from baseline compared with the manual acupuncture group (P <.001). No significant differences in the changes in the mean MMST and EQ-5D scores were found between the two groups (P >.05) (Table 2).

**Figure 2:**
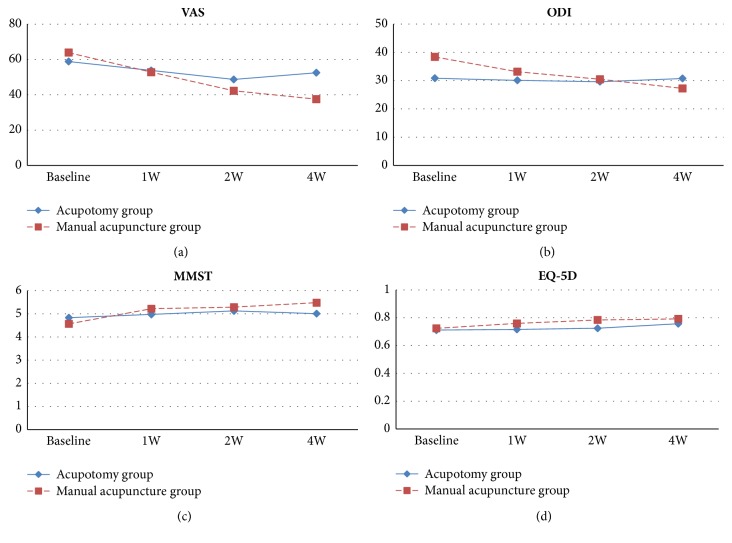
The time × group interaction effect on VAS, ODI, MMST, and EQ-5D.

To investigate if the time × group interaction effect on outcome measures is significant, a repeated measures ANOVA was performed using the measurements obtained at the 1st, 2nd, and 4th weeks. For accurate results, we first investigated whether the variables satisfied the assumption of sphericity before performing the repeated measures ANOVA. The VAS and ODI scores satisfied the assumption of sphericity, and MMST and EQ-5D scores did not satisfy the assumption of sphericity. The statistical significance of the trends in the VAS, ODI, MMST, and EQ-5D scores at the 1st, 2nd, and 4th weeks was presented in Figure 2. The change was significant in VAS score (P <.01) and ODI score (P <.001) but not in MMST and EQ-5D score.

### 3.4. Safety of Acupuncture

Seven participants reported a total of nine adverse events. There were two adverse events in two participants, which were thought to be related to this clinical trial. Two cases were observed in the acupotomy group. In one case, a participant became dizzy after undergoing the first acupotomy session and recovered the day after. The participant underwent the subsequent acupotomy sessions as scheduled, not feeling dizzy. The second case included delirium, dizziness, nausea, and vomiting, which was considered to have resulted from lidocaine use [24]. The participant was withdrawn from this trial, although recovering without any sequela. The other seven cases included premenstrual disorder, common cold, and stomach ache, which were not related to this trial.

## 4. Discussion

This study was designed to investigate the clinical effectiveness of acupotomy compared with manual acupuncture and the safety of both interventional methods. The VAS score decreased more significantly in the acupotomy group, in which participants received four acupotomy sessions for 2 weeks, than in manual acupuncture group, in which participants received six manual acupuncture sessions for 2 weeks, suggesting a better therapeutic effect on relieving the pain caused by LDH. After acupotomy, MMST, ODI, and EQ-5D were also improved significantly, and more significant changes were shown in the ODI compared with the manual acupuncture. The VAS and ODI decreased constantly as the acupotomy sessions went on, which can be interpreted as the fact that the improvement of pain and disability is not temporary but consistent.

The concept of acupotomy existed in ancient times, but the modern form of acupotomy was first introduced by a Chinese professor named Hanzhang Zhu in 1975 [25]. Acupotomy needle possesses greater diameters than a manual acupuncture needle and can be applied not only on traditional acupoints but also on musculotendinous junctions in contact with muscles, tendons, and bones [26]. Acupotomy can recover the dynamic function of soft tissues, relieve abnormal pressure applied on nerves, and promote Qi-blood circulation to relieve pain [27]. These acupotomy mechanisms have been utilized in the treatment of various disorders [11–17]. There have been extensive researches on appropriate methods for LDH, which can induce chronic pain and reduce the quality of life (QOL) [28], and acupotomy is thought to be another option. In clinical practice, we have applied acupotomy on patients with lumbar spinal disorders including herniated disc and stenosis and observed satisfactory therapeutic effect.

In this study, acupotomy produced superior results compared to manual acupuncture, possibly because of the following reasons. First, the needles used in acupotomy could restore the biodynamic balance at the lumbar spine by releasing unnecessary tension in deep muscles, recovering muscle strength, and reducing muscle fatigue to mitigate the lumbar extensor muscle imbalances [16]. Second, acupotomy may be more effective than manual acupuncture in that acupotomy can ameliorate the nerve entrapment physically, as well as promote the circulation of a lesion. When a lumbar nerve root gets compressed by fibrous tissues, the adhesion can cause low-back pain and/or radiating pain [29]. Furthermore, local adhesion disrupts the blood flow, and when it comes to the circulation to nerves, the symptoms above can be worse [30]. In another point, because LDH is associated with the atrophy of the paraspinal muscles [31], acupotomy may help to recover the atrophy by relieving the nerve root entrapment indirectly. Third, we can explain the therapeutic effects of acupotomy with a stronger stimulus than manual acupuncture procedure. By directly applying a strong stimulus at the painful spot with an acupotomy needle, the pain threshold can be lowered and the signals that affect pain perception can be reduced [32].

The present study has several limitations. First, the population of recruited participants was small. Second, the follow-up period was short. Lastly, the effects of manual acupuncture were not very evident. This result might be due to short intervention period and weak stimulation made by thin needle compared with acupotomy. It would be useful for future studies to consider monitoring more participants for a longer term, increasing the number of treatment sessions, and using larger acupuncture needles.

## 5. Conclusion

The present results indicated a significant difference in the intensity of pain and disability in patients with LDH after 4 times of acupotomy compared with 6 times of manual acupuncture for 2 weeks. The ability to reduce the pain and disability continued significantly as the acupotomy treatment was repeated suggesting that multiple acupotomy treatments can relieve pain and disability caused by LDH effectively.

## Figures and Tables

**Table 1:** Baseline VAS, MMST, ODI, and EQ-5D between two groups.

	Acupotomy group (n = 25)	Manual acupuncture group (n = 25)	p-value
VAS	63.88 ± 16.599	60.60 ± 17.260	.497
MMST	4.57 ± 1.774	4.66 ± 1.566	.853
ODI	38.40 ± 12.657	32.37 ± 14.190	.120
EQ-5D	0.78 ± 0.117	0.72 ± 0.118	.115

M, mean; SD, standard deviation; VAS, Visual Analogue Scale, MMST, Modified-Modified Schober Test; OD, Oswestry Disability Index; EQ-5D questionnaire, EuroQol Five Dimensions questionnaire.

**Table 2:** Observed outcomes and p-value of adjusted group differences in the analysis of covariance.

Variable	Week	Acupotomy group (n=25)	Manual acupuncture group (n=25)	Adjusted mean difference	p-value
VAS	Baseline	62.74 (16.666)	58.83 (16.846)	18.56	.002
	4	37.52 (24.714)	52.48 (23.216)		

MMST	Baseline	4.69 (1.786)	4.84 (1.348)	0.55	.129
	4	5.48 (1.581)	5.01 (1.304)		

ODI	Baseline	36.81 (11.678)	30.84 (13.641)	8.56	<.001
	4	27.24 (12.302)	30.76 (14.078)		

EQ-5D	Baseline	0.779 (0.121)	0.737 (0.112)	.005	.458
	4	0.792 (0.114)	0.757 (0.111)		

Observed outcomes are presented as mean (SD).

VAS: Visual Analogue Scale; MMST: Modified-Modified Schober Test; ODI: Oswestry Disability Index; EQ-5D questionnaire: EuroQol Five Dimensions questionnaire.

## Data Availability

All the outcome values were registered on the eCRF, which was designed by Korea Institute of Oriental Medicine.
