# Corrigendum to “Effectiveness and Safety of Acupotomy for Lumbar Disc Herniation: A Randomized, Assessor-Blinded, Controlled Pilot Study”

**DOI:** 10.1155/2019/4538692

**Published:** 2019-04-03

**Authors:** So Yun Kim, Eunseok Kim, Ojin Kwon, Chang-Hyun Han, Young-Il Kim

**Affiliations:** ^1^Department of Acupuncture and Moxibustion Medicine, Daejeon University Dunsan Korean Medicine Hospital, Daejeon 35235, Republic of Korea; ^2^Department of Acupuncture & Moxibustion Medicine, College of Korean Medicine, Daejeon University, 62 Daehak-ro, Dong-gu, Daejeon 34520, Republic of Korea; ^3^Clinical Research Division, Korea Institute of Oriental Medicine, Daejeon 34054, Republic of Korea

In the article titled “Effectiveness and Safety of Acupotomy for Lumbar Disc Herniation: A Randomized, Assessor-Blinded, Controlled Pilot Study” [1], there was an error in the key of Figure 2, where the blue lines should be “Manual acupuncture group” and the red lines should be “Acupotomy group”. The correct figure is shown below.

## Figures and Tables

**Figure 2:**
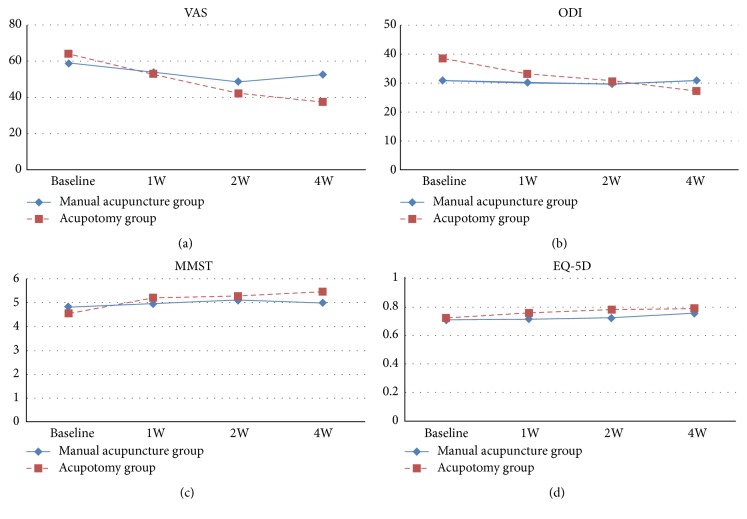
The time × group interaction effect on VAS, ODI, MMST, and EQ-5D.
